# Supplementary material for: Clinical implications of the log linear association between LDL-C lowering and cardiovascular risk reduction: Greatest benefits when LDL-C >100 mg/dl
Source: PLoS One. 2020 Oct 29;15(10):e0240166. doi: 10.1371/journal.pone.0240166 (PMC7595281; doi:10.1371/journal.pone.0240166)
Supplement: S1 Fig — (RTF) [file pone.0240166.s001.rtf]

S1 Fig. Rate of incident MACE per 100 patient years by average achieved on-treatment LDL-D levels in patients with ASCVD with and without comorbidities in a pooled analysis of alirocumab Phase 3a trials using a multivariate Poisson analysis adjusted for baseline characteristics
From: Vallejo-Vaz AJ, Ray KK, Ginsberg HN, et al. Associations between lower levels of low-density lipoprotein cholesterol and cardiovascular events in very high-risk patients: Pooled analysis of nine ODYSSEY trials of alirocumab. Atherosclerosis. 2019;288:85-93.
