# Supplementary material for: Clinical implications of the log linear association between LDL-C lowering and cardiovascular risk reduction: Greatest benefits when LDL-C >100 mg/dl
Source: PLoS One. 2020 Oct 29;15(10):e0240166. doi: 10.1371/journal.pone.0240166 (PMC7595281; doi:10.1371/journal.pone.0240166)
Supplement: S2 Fig — (RTF) [file pone.0240166.s002.rtf]

S2 Fig. Reductions in the risk of major cardiovascular events per 39 mg/dl (1 mmol) reduction in LDL-C in trials of moderate versus high intensity statin therapy


CTT Collaborators. Cholesterol Treatment Trialists' (CTT) Collaboration web slide deck 08-30-2016: Effect of statin therapy on MAJOR VASCULAR EVENTS:5 trials of more vs. less statin. 2016 December 20, 2017]; Available from: https://www.cttcollaboration.org/efficacy-web-page
