# Supplementary material for: Clinical implications of the log linear association between LDL-C lowering and cardiovascular risk reduction: Greatest benefits when LDL-C >100 mg/dl
Source: PLoS One. 2020 Oct 29;15(10):e0240166. doi: 10.1371/journal.pone.0240166 (PMC7595281; doi:10.1371/journal.pone.0240166)
Supplement: S3 Fig — (RTF) [file pone.0240166.s003.rtf]

S3 Fig. Sensitivity analysis of the MACE primary outcome for subgroups from the ODYSSEY OUTCOMES alirocumab cardiovascular outcomes trial fitted as log linear association weighted by group size


MACE	Major adverse cardiovascular events defined as coronary heart disease death, nonfatal 
	myocardial infarction, fatal or nonfatal stroke, or hospitalized unstable angina
* On-treatment LDL-C levels were not reported fro these subgroup analyses; Mean LDL-C levels were estimated from figures is available, or calculated from the information on baseline and expected 2-year LDL-C level and mean percent LDL-C reduction

Reference
Schwartz GG, Steg PG, Szarek M, Bhatt DL, Bittner VA, Diaz R, Edelberg JM, Goodman SG, 
Hanotin C, et al.  Alirocumab and Cardiovascular Outcomes after Acute Coronary Syndrome. N Engl J Med 2018; 379: 2097-2107.
