# Supplementary material for: Clinical implications of the log linear association between LDL-C lowering and cardiovascular risk reduction: Greatest benefits when LDL-C >100 mg/dl
Source: PLoS One. 2020 Oct 29;15(10):e0240166. doi: 10.1371/journal.pone.0240166 (PMC7595281; doi:10.1371/journal.pone.0240166)
Supplement: S4 Fig — (RTF) [file pone.0240166.s004.rtf]

S4 Fig. Sensitivity analysis using a 25% reduction in the relative risk of ASCVD over 5 years of therapy in subgroups of the FOURIER evolocumab cardiovascular outcomes trial fitted as log linear association weighted by group size
