# Supplementary material for: Clinical implications of the log linear association between LDL-C lowering and cardiovascular risk reduction: Greatest benefits when LDL-C >100 mg/dl
Source: PLoS One. 2020 Oct 29;15(10):e0240166. doi: 10.1371/journal.pone.0240166 (PMC7595281; doi:10.1371/journal.pone.0240166)
Supplement: S1 File — (PDF) [file pone.0240166.s006.pdf]

METHODS

Systematic review of randomized, controlled cardiovascular outcomes trials comparing moderate to high intensity statin therapy and PCSK9 inhibiting monoclonal antibodies

Randomized control trials (RCTs) and subgroup analyses of the RCTs with ASCVD endpoints were identified in a previous systematic review (Robinson, J.G., et al., *Determining When to Add Nonstatin Therapy: A Quantitative Approach.* Journal of the American College of Cardiology, 2016. **68**(22): 2412-2421), which included cardiovascular outcomes trials comparing high versus moderate intensity statins. These data were supplemented with a systematic review of trials of drugs approved by the Food and Drug Administration for clinical use that were published subsequent to April 2016- trials of PCSK9 inhibitor versus placebo in patients treated with high or moderate intensity statin therapy. Only trials reporting ASCVD rates defined as cardiovascular or coronary heart disease, non-fatal myocardial infarction, and fatal or non-fatal stroke were included. Trials including majority of patients with heart failure or end-stage renal disease were excluded.

Search strategy

The search strategy and inclusion criteria used for the systematic review performed for the Robinson, et al 2016 paper were repeated using inclusion dates of 4/7/2016 through 10/30/19. The endpoint of interest was composite ASCVD, which included myocardial infarction, stroke and cardiovascular death (or coronary heart disease or all-cause death if cardiovascular death not available).

To identify trials with ASCVD outcomes, MEDLINE/PubMed was searched by generic drug name, "cardiovascular", "randomized, controlled trial", and their combinations as search terms and subsequently used the trials reporting these events to identify subgroup analyses of patients with diabetes, chronic kidney disease (CKD), LDL-C $\geq$190 mg/dl, lipoprotein (a), recurrent cardiovascular events, acute coronary syndromes, elevated CRP, peripheral vascular disease or other subgroups. The author's files were hand-searched to identify any additional subgroup analyses from meeting abstracts or publications. Inclusion dates were 4/7/2016 through 6/29/19. In addition, we searched Clinical trials.gov for RCTs with available results which had been published in peer reviewed journals. No language restriction was used.

Subgroup analyses were included regardless of whether they were pre-specified in the protocol or *post hoc*. All subgroup analyses came from double-blind placebo-controlled randomized controlled cardiovascular outcomes trials with the exception of the IDEAL trial which was a open-label PROBE design. All trials had pre-specified endpoints and independent event adjudication. All trials were funded by pharmaceutical companies. All trials were funded by pharmaceutical companies with oversight of independent steering committee. IMPROVE-IT, FOURIER and ODYSSEY OUTCOMES had an independent group perform the trial and collect and analyze data. Risk of bias for all subgroup analyses was considered low.

Trial data were abstracted and confirmed by 2 authors (JGR and MBJ).

Statistical methods

The mean or median LDL-C levels for each treatment group after 1 year of treatment were used if available, otherwise LDL-C levels as close to mid-trial as possible were used.

The annualized rate was calculated as the observed rate for the duration of the trial divided by the median/mean trial duration or Kaplan-Meier (KM) follow-up period as available. The 5-year absolute ASCVD risks were estimated for each treatment group by multiplying the annualized rate observed during the trial by 5.

The subgroups were then classified into risk groups >4.0%, 3.0-3.9%, and <3.0% based on annualized ASCVD absolute risk in placebo arms in PCSK9 and ezetimibe trials and moderate intensity statin arms of the statin trials.

Least-squares regression weighted by sample size for each subgroup was used to characterize the relationship between mean on-treatment LDL cholesterol and absolute ASCVD rates for various risk groups. A log-linear model Ln (Event rate) =LDL-C (milligrams per deciliter) was fit for each risk group for statin and PCSK9 trials individually.

All statistical analyses were performed using the Statistical Analysis System (SAS) statistical software package, version 9.4. SAS Institute Inc., Cary, NC, USA.

Sensitivity analysis

Since PCSK9 trials reported an increasing magnitude of the relative reduction in ASCVD risk from 16% (95% CI,4 to 26) in the first year to 25% (95% CI, 11 to 27) in the second year, a sensitivity analysis was performed using an annualized rate based on a 25% relative risk reduction in ASCVD in the evolocumab compared to placebo group.
