# Supplementary material for: Clinical implications of the log linear association between LDL-C lowering and cardiovascular risk reduction: Greatest benefits when LDL-C >100 mg/dl
Source: PLoS One. 2020 Oct 29;15(10):e0240166. doi: 10.1371/journal.pone.0240166 (PMC7595281; doi:10.1371/journal.pone.0240166)
Supplement: S2 File — (PDF) [file pone.0240166.s007.pdf]

SYSTEMATIC REVIEW RESULTS

Moderate versus high intensity statin trial subgroups

No new publications were identified that reported subgroup analyses with ASCVD outcomes for the moderate versus high intensity statin trials. The data included below are from Robinson, J.G., et al., *Determining When to Add Nonstatin Therapy: A Quantitative Approach.* Journal of the American College of Cardiology, 2016. **68**(22): 2412-2421 were used for the analyses of moderate versus high intensity statins in this paper.

Data Table A - Moderate versus high intensity statin trial subgroups

All participants had coronary heart disease. Subjects were randomized to a high intensity statin (atorvastatin 80 mg) or moderate intensity statin (atorvastatin 10 mg or simvastatin 20-40 mg).

Trial	Statin intensity	On-treatment LDL-C	Median trial duration	Observed rate for trial	Annualized rate
**≥4.0% annualized ASCVD risk**					
IDEAL Simv PVD	Moderate	104	4.8	26.3	5.5
IDEAL Ato PVD	High	86	4.8	13.1	4.3
TNT 10 DM CKD	Moderate	99	4.9	20.9	4.3
TNT 80 DM CKD	High	75	4.9	13.9	2.8
TNT 10 resistant HTN	Moderate	99	4.9	19.9	4.1
TNT 80 resistant HTN	High	77	4.9	14.5	3.0
**3.0-<4% annualized ASCVD risk**					
TNT/IDEAL 10 smoker	Moderate	99	4.9	15.2	3.6
TNT/IDEAL 80 smoker	High	77	4.9	13.6	2.8
IDEAL CKD simv	Moderate	100	4.8	16.9	3.5
IDEAL CKD ato	High	80	4.8	16.1	3.4
TNT 10 DM	Moderate	99	4.9	17.9	3.7
TNT 80 DM	High	77	4.9	13.8	2.8
IDEAL 65-80 Simv	Mod	99	4.8	17.4	3.6
IDEAL 65-80 Ato	High	83	4.8	16.2	3.4
**<3.0% annulaized ASCVD risk**					
TNT + MS 10	Moderate	99	4.9	11.6	2.4
TNT + MS 80	High	73	4.9	8.2	1.7
TNT-No MS 10	Moderate	99	4.9	9.1	2.0
TNT-No MS 80	High	73	4.9	8.1	1.6
TNT 10-No DM	Moderate	99	4.9	9.7	2.0
TNT 80-No DM	High	77	4.9	7.8	1.6
TNT-No res HTN 10	Moderate	99	4.9	9.8	2.0
TNT-No res HTN 80	High	79	4.9	8.0	1.6
IDEAL no CKD sim	Moderate	100	4.8	12.5	2.6
IDEAL no CKD ato	High	80	4.8	10.5	2.2

TNT CHD + CABG 10	Moderate	101	4.9	13.0	2.7
TNT CHD + CABG 80	High	79	4.9	97.0	2.0
IDEAL -Ato<65 years	High	83	4.8	8.9	1.9
IDEAL -Sim<65 years	Moderate	104	4.8	11.0	2.3
IDEAL-Ato-no PVD	High	82	4.8	11.6	2.4
IDEAL-Simv-no PVD	Moderate	101	4.8	13.1	2.7
IDEAl/TNT fm smk Sim	Moderate	100	4.9	12.9	2.6
IDEAL/TNt Nv Smk sim	Moderate	100	4.9	11.8	2.4
IDEAl/TNT fm smk Ato 10	Moderate	99	4.9	10.7	2.2
IDEAl/TNT Nv smk Ato 10	Moderate	99	4.9	9.3	1.9
IDEAl/TNT fm smk Ato 80	High	77	4.9	9.6	2.0
IDEAl/TNT Nv smk Ato 80	High	77	4.9	9.7	2.0
TNT 65-75 10	Moderate	97	4.9	12.6	2.57
TNT 65-75	High	72	4.9	10.3	2.10
TNT<65 10	Moderate	97	4.9	10	2.041
TNT<65 80	High	72	4.9	7.7	1.571
TNT DM no CKD 10	Moderate	98.6	4.9	14.1	2.877
TNT DM no CKD 80	High	74.9	4.9	12.8	2.612

Abbreviations

LDL	Mean/Median on treatment LDL cholesterol in mg/dL
AR	Estimated 10-year Absolute Risk %
n	Sample size in each sub group
LogRisk	Natural log of Extrapolated 10-year Absolute Risk %
Predicted Risk	Predicted 10-year Absolute Risk by the Log Linear model.

Abbreviations
Statin trial subgroups

IDEAL	Incremental Decrease in End Points Through Aggressive Lipid Lowering (IDEAL) Study Group.
TNT	Treating to New Targets (TNT) trial
Ato	Atorvastatin
Simv	Simvastatin
DM	Diabetes Mellitus
MS	Metabolic Syndrome
CABG	Coronary artery bypass graft
res HTN	Resistant Hypertension
CKD	Chronic kidney disease defined as eGFR <60 mL/min/1.73 m2
PVD	Peripheral arterial disease
fm smk	Former Smoker
Nv smk	Never Smoker
CHD	Coronary Heart Disease

<65	Age less than 65 years
65-80	Age 65 years to 80 years
65-75	Age 65 to 75 years
10	Atorvastatin dose 10mg/day
80	Atorvastatin dose 80mg/day

Ezetimibe versus placebo with background statin therapy

An updated PubMED search was performed on 10/30/10including the last name of the IMPROVE-IT principal investigator (Cannon) and limited to Jan 1, 2015 or later. The results of the search are provided in **Supplemental Figure A and Data Table B** below. One trial of ezetimibe was identified, IMPROVE-IT. This trial enrolled subjects with a recent acute coronary syndrome who had an LDL-C 50-100 mg/dl on simvastatin therapy, or 50-120 mg/dl if not on statin therapy.

Several subgroup analyses have been published, but only 3 analyses reported ASCVD event rates for the subgroups.

Subgroups identified:

- High risk (≥3 risk enhancers)[1]
- Intermediate risk (2 risk enhancers)[1]
- Low risk (0-1 risk enhancer)[1]
    - Risk enhancers: Heart failure, hypertension, age ≥75 years, diabetes mellitus, prior stroke, prior coronary artery bypass grafting, peripheral arterial disease, estimated glomerular filtration rate <60 ml/min/1.73 m^2, current smoking)
- Diabetes[2]
- No diabetes[2]
- Age ≥75 years[3]
- Age 65-74 years[3]
- Age <65 years[3]

Supplemental Figure A

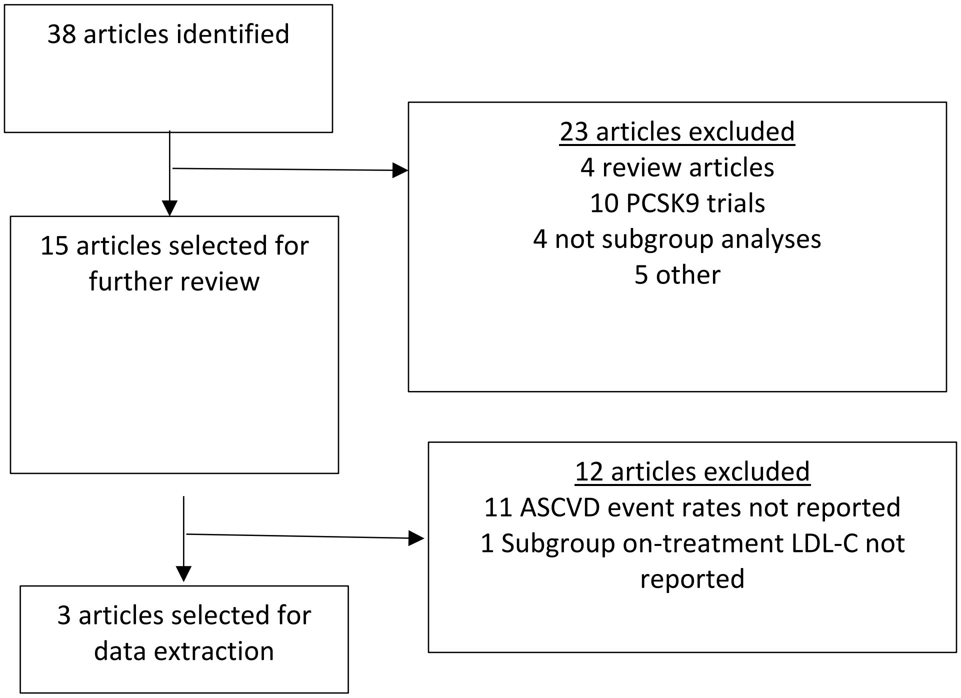

Data Table B. Ezetimibe cardiovascular outcomes trial

Trial subgroup	Randomized treatment	On-treatment LDL-C	KM trial duration	Observed rate for trial	Annualized rate
**Risk >=40%**					
IMPROVE-IT >3 HRI	Ezetimibe	48	7	33.9	4.8
IMPROVE-IT >3 HRI	Simvastatin	66	7	40.2	5.7
IMPROVE-IT 75	Ezetimibe	46	7	31.3	4.5
IMPROVE-IT 75	Simvastatin	64	7	38	5.4
IMPROVE-IT DM	Simvastatin	65	7	29.9	4.3
IMPROVE-IT DM	Ezetimibe	46	7	25.3	3.6
**Risk30-39%**					0.0
IMPROV-IT 2 HRI	Ezetimibe	49	7	19.3	2.8
IMPROVE-IT 2 HRI	Simvastatin	67	7	21.5	3.1
IMPROVE-IT 65-74	Ezetimibe	48	7	22.4	3.2
IMPROVE-IT 65-74	Simvastatin	66	7	23	3.3
**Risk 20-29%**					0.0
IMPROVE-IT 0-1 HRI	Ezetimibe	51	7	13.1	2.0
IMPROVE-IT 0-1 HRI	Simvastatin	68	7	14	1.9

IMPROVE-IT <65	Ezetimibe	51	7	16.6	2.4
IMPROVE-IT <65	Simvastatin	69	7	17.8	2.5
IMPROVE-IT no DM	Ezetimibe	46	7	17.2	2.5
IMPROVE-IT no DM	Simvastatin	65	7	18	2.6

References

1. Bohula EA, Morrow DA, Giugliano RP, et al. Atherothrombotic Risk Stratification and Ezetimibe for Secondary Prevention. *J Am Coll Cardiol.* 2017;69(8):911-921.
2. Giugliano RP, Cannon CP, Blazing MA, et al. Benefit of Adding Ezetimibe to Statin Therapy on Cardiovascular Outcomes and Safety in Patients With Versus Without Diabetes Mellitus*. Circulation* 2018;137(15):1571-1582.
3. Bach RG, Cannon CP, Giugliano RP, et al. Effect of Simvastatin-Ezetimibe Compared With Simvastatin Monotherapy After Acute Coronary Syndrome Among Patients 75 Years or Older: A Secondary Analysis of a Randomized Clinical Trial. *JAMA Cardiology.* 2019; 4: 846-854

PCSK9 inhibitors versus placebo on background statin therapy

The results of the search are provided in **Supplemental Figure B & Data Table C** below. The evolocumab trial FOURIER enrolled subjects with chronic ASCVD and additional high risk characteristics. All were treated with moderate or high intensity statin therapy and had an LDL-C ≥70 mg/dl or non-HDL-C ≥100 mg/dl at baseline (1). ASCVD was a secondary outcome.

The alirocumab trial ODYSSEY OUTCOMES trial enrolled subjects with an acute coronary syndrome <1 year prior to screening. All were treated with moderate or high intensity statin therapy and had an LDL-C ≥70 mg/dl or non-HDL-C ≥100 mg/dl at baseline (2). No ASCVD outcome (defined as CVD death, myocardial infarction, or stroke) was reported for this trial.

Subgroups identified in FOURIER:

- ASCVD stratified by hsCRP levels <1mg/dL, 1 to 3 mg/DL, > 3mg/dL.[1]
- ASCVD with history of myocardial infarction, presence and absence of Peripheral Arterial disease.[2]
- ASCVD on background maximal statin therapy and submaximal therapy.[3]
- ASCVD with baseline LDL less than 70mg/dL and more than 70mg/dL.[3]
- ASCVD with more than 2 prior myocardial infarction episodes and less than 2 prior myocardial infarctions.[4]
- ASCVD with recent myocardial infarction (< 2 years) and with history of myocardial infarction more than 2 years ago. [4]
- ASCVD with multi-vessel coronary artery disease and without multi-vessel coronary artery disease.[4]
- ASCVD with diabetes mellitus and without diabetes mellitus.[5]
- ASCVD stratified by Lp(a) <37 nM or ≥37 nM[8]

Supplemental Figure B

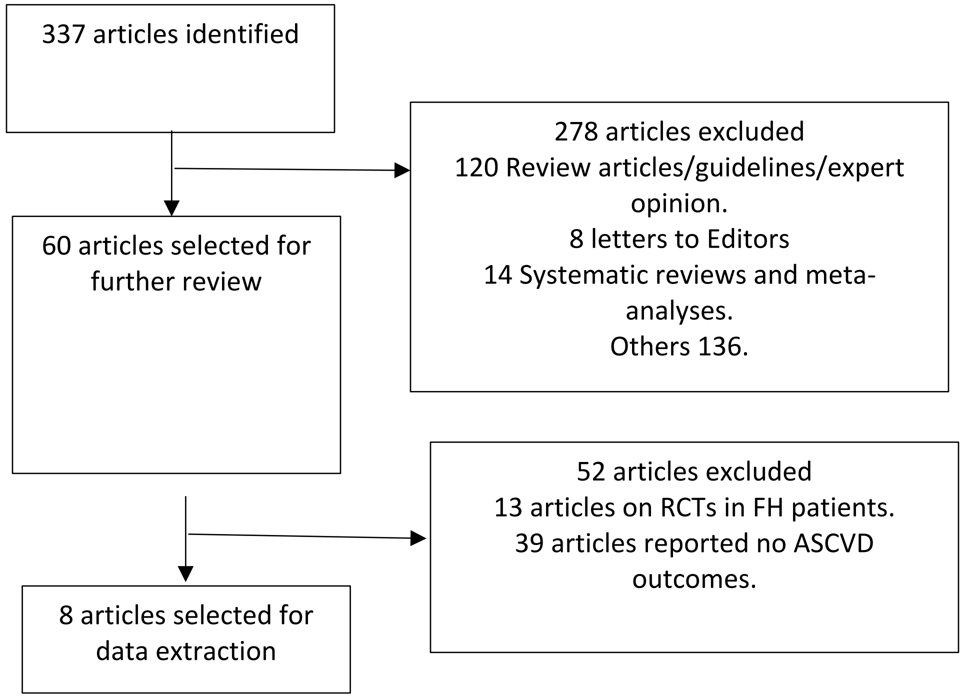

Data Table C. PCSK9 inhibitor trial subgroups

All participants have cardiovascular disease and were receiving background statin therapy

Trial subgroup	Randomized treatment	On-treatment LDL-C	Median or KM Trial Duration	Observed Rate for trial	Annualized rate
**≥4.0% annualized ASCVD risk**					
Fourier PVD MI CVA Placebo	Placebo	94	2.5	14.9	5.2
Fourier PVD MI CVA PCKS9	PCSK9	31	2.5	9.5	3.8
Fourier 2PRIORMI Placebo	Placebo	92	3	15	5.0
Fourier 2PRIORMI PCSK9	PCSK9	30	3	12.4	4.1
Fourier MVD Placebo	Placebo	93	3	12.6	4.2
Fourier MVD PCKS9	PCSK9	30	3	9.2	3.1
Fourier No max Statin placebo	Placebo	91	2.2	9.7	4.4
Fourier No max Statin pcsk9	PCSK9	32	2.2	7.9	3.6
ODYSSEY Placebo	Placebo	101.4	2.8	11.9	4.3
ODYSSEY PCSK9	PCSK9	53.3	2.8	10.3	3.7
Fourier hsCRP>3 Placebo	Placebo	94	3	13.2	4.4
Fourier hsCRP>3 PCSK9	PCSK9	30	3	10.2	3.4
Fourier DM Placebo	Placebo	89	3	12.2	4.1
Fourier DM PCSK9	PCSK9	31	3	10.2	3.4

**3.0-3.9% annualized ASCVD risk**					
Fourier Recent MI Placebo	Placebo	90	3	10.8	3.6
Fourier Recent MI PCSK9	PCSK9	29	3	7.9	2.6
Fourier no Recent MI Placebo	Placebo	93	3	9.3	3.1
Fourier no Recent MI PCSK9	PCSK9	30	3	8.3	2.8
Fourier MI CVA no PVD Placebo	Placebo	91	2.5	7.6	3.0
Fourier MI CVA no PVD	PCSK9	31	2.5	6.2	2.5
Fourier Max Statin placebo	Placebo	93	2.2	8.3	3.8
Fourier Max Statin pcsk9	PCSK9	32	2.2	6.5	3.0
Fourier <70 placebo	Placebo	65.5	2.2	6.8	3.1
Fourier <70 pcsk9	PCSK9	21	2.2	4.7	2.1
Fourier>70 placebo	Placebo	93.5	2.2	7.4	3.4
Fourier>70 pcsk9	PCSK9	32	2.2	6	2.7
Fourier hsCRP 1 to 3 Placebo	Placebo	92	3	9.1	3.0
Fourier hsCRP 1 to 3 PCSK9	PCSK9	30	3	7.1	2.4
Fourier Lp(a) >=37 nM	Placebo	98	3	11	3.7
Fourier Lp(a) >=37 nM	PCSK9	30	3	8.2	2.8
**<30% 10-year ASCVD risk**					
Fourier No DM Placebo	Placebo	93	3	8.4	2.8
Fourier No DM PCSK9	PCSK9	31	3	6.4	2.1
Fourier no MVD Placebo	Placebo	92	3	8.9	3.0
Fourier no MVD PCSK9	PCSK9	29	3	7.6	2.5
Fourier <2PRIORMI Placebo	Placebo	92	3	8.2	2.7
Fourier <2PRIORMI PCKS9	PCSK9	29	3	6.6	2.2
Fourier hsCRP<1 Placebo	Placebo	90	3	7.4	2.5
Fourier hsCRP<1 PCSK9	PCSK9	30	3	6.6	2.2
Fourier Lp(a) <37 nM	Placebo	95	3	8.7	2.9
Fourier Lp(a) <37 nM	PCSK9	25	3	7.5	2.5

Abbreviations PCSK9 trials

**FOURIER**	Further Cardiovascular Outcomes Research with PCSK9 Inhibition in Subjects with Elevated Risk
**ODYSSEY**	Evaluation of Cardiovascular Outcomes After an Acute Coronary Syndrome During Treatment With Alirocumab
**PVD**	Peripheral vascular disease
**CVA**	Cerebrovascular accident
**MI**	Myocardial Infarction
**<2PRIORMI**	One prior myocardial infarction episode
**2PRIORMI**	2 or more prior myocardial infarction events
**MVD**	Presence of residual Multivessel Coronary artery disease (≥40% stenosis in ≥2 major vessels)
**No MVD**	Absence of residual Multivessel Coronary artery disease (≥40% stenosis in ≥2 major vessels)
**No DM**	No known history of Diabetes Mellitus
**DM**	Diabetes Mellitus
**hsCRP<1**	Baseline high-sensitivity C-reactive protein < 1mg/dL
**hsCRP<1 to 3**	Baseline high-sensitivity C-reactive protein 1-3 mg/dL

**hsCRP>3**	Baseline high-sensitivity C-reactive protein >3 mg/dL
**Recent MI**	Qualifying myocardial infarction equal to or more than 2 years ago
**no Recent MI**	Qualifying myocardial infarction less than 2 years ago
**<70**	Baseline LDL-C <70 mg/dL
**>70**	Baseline LDL-C >70 mg/dL
**Max Statin**	Maximal Potency Background Statin
**No max Statin**	Submaximal Potency Background Statin
**MI CVA no PVD**	History of prior Myocardial infarction or prior stroke and no known peripheral arterial disease
**PVD MI CVA**	History of prior Myocardial infarction or prior stroke and concomitant peripheral arterial disease
**LDL**	mean on treatment LDL cholesterol in mg/dL
**Lp(a)**	Lipoprotein (a)

References

1. Bohula EA, Giugliano RP, Leiter LA, et al. Inflammatory and Cholesterol Risk in the FOURIER Trial (Further Cardiovascular Outcomes Research With PCSK9 Inhibition in Patients With Elevated Risk). *Circulation.* 2018; 138: 131-140.
2. Bonaca MP, Nault P, Giugliano RP, et al. Low-Density Lipoprotein Cholesterol Lowering With Evolocumab and Outcomes in Patients With Peripheral Artery Disease: Insights From the FOURIER Trial (Further Cardiovascular Outcomes Research With PCSK9 Inhibition in Subjects With Elevated Risk). *Circulation.* 2018;137(4):338-350.
3. Giugliano RP, Keech A, Murphy SA, et al. Clinical Efficacy and Safety of Evolocumab in High-Risk Patients Receiving a Statin: Secondary Analysis of Patients With Low LDL Cholesterol Levels and in Those Already Receiving a Maximal-Potency Statin in a Randomized Clinical Trial. *JAMA Cardiology.* 2017;2(12):1385-1391.
4. Sabatine MS, De Ferrari GM, Giugliano RP, et al. Clinical Benefit of Evolocumab by Severity and Extent of Coronary Artery Disease: An Analysis from FOURIER. *Circulation.* 2018; 138: 756-766.
5. Sabatine MS, Leiter LA, Wiviott SD, et al. Cardiovascular safety and efficacy of the PCSK9 inhibitor evolocumab in patients with and without diabetes and the effect of evolocumab on glycaemia and risk of new-onset diabetes: a prespecified analysis of the FOURIER randomised controlled trial. *Lancet Diab Endocrinol.* 2017;5(12):941-950.
6. Giugliano RP, Pedersen TR, Park JG, et al. Clinical efficacy and safety of achieving very low LDL-cholesterol concentrations with the PCSK9 inhibitor evolocumab: a prespecified secondary analysis of the FOURIER trial. *Lancet.* 2017;390(10106):1962-1971.
7. Sabatine MS, Giugliano RP, Keech AC, et al. Evolocumab and Clinical Outcomes in Patients with Cardiovascular Disease. *N Engl J Med.* 2017;376(18):1713-1722.
8. O'Donoghue M, Fazio S, Giugliano R, et al. Lipoprotein(a), PCSK9 Inhibition, and Cardiovascular Risk. *Circulation.* 2019;139(12):1483-1492.
